# Indirect Influence of African Swine Fever Outbreak on the Raven (*Corvus corax*) Population

**DOI:** 10.3390/ani9020041

**Published:** 2019-01-30

**Authors:** Jakub Gryz, Dagny Krauze-Gryz

**Affiliations:** 1 Department of Forest Ecology, Forest Research Institute, Sękocin Stary, Braci Leśnej 3, 05-090 Raszyn, Poland; 2 Department of Forest Zoology and Wildlife Management, Faculty of Forestry, Warsaw University of Life Sciences, Nowoursynowska 159, 02-776 Warszawa, Poland; dagny.krauze@wl.sggw.pl

**Keywords:** Scavenger, carrion availability, Central Poland, field and forest mosaic, population density, breeding parameters

## Abstract

**Simple Summary:**

The persecution of ravens ceased in the middle of the 20th century, and consequently their abundance started to rise in most European countries. Ravens are food opportunists that can eat plants, prey on animals, and feed on garbage dumps. However, carrion plays a crucial role in their diet. In Central Poland, the carrion of farm animals (disposed of illegally) was thought to be the main food source for ravens. This changed radically in February 2014, after the first case of African swine fever (ASF) in Poland, and the introduction of strict procedures for the utilization of pig and wild boar carcasses to stop the spread of the virus. This decrease in carrion availability influenced the raven population; ravens changed their diet, i.e., instead of feeding on the carrion of pigs (previously their main dietary component), they ate (among others) the carrion of domestic dogs and cats, and also preyed on small vertebrates. Consequently, the number of breeding raven pairs decreased, and birds produced fewer fledglings. This research showed how certain human activities influence wildlife. The illegal disposal of dead animals, motivated by the desire to save money, resulted in abundant food sources for ravens and an increase in population size.

**Abstract:**

Carrion plays a crucial role in the raven’s diet. In the past, domestic pig carrion was widely available in Poland. This changed with an African swine fever (ASF) outbreak and the introduction of strict procedures aimed at stopping the virus from spreading. We compared data from Central Poland (field and forest mosaic, study area of 105 km^2^) for two periods, i.e., before (2011–2014) and after the ASF outbreak (2015–2018). In breeding seasons, nests of ravens were found, juveniles were counted, and the time when juveniles left their nests was recorded. Diet composition data were based on pellet analysis and direct observations of feeding birds. The number of breeding pairs dropped from 12.3 to 7.5 in the second period. Breeding parameters were similar. However, birds in the second period had fewer fledglings per successful pair. Domestic pig carrion was found to be an important food item, and with its limited supply, ravens changed their diet, i.e., they fed on the carrion of dogs and cats or preyed on small vertebrates more often. Overall, our study points to a crucial role of the availability of the carrion of big farm animals (i.e., domestic pig) in maintaining the high density of breeding raven populations.

## 1. Introduction

The persecution of ravens (*Corvus corax*) ceased in the middle of the 20th century, and consequently the abundance of the species started to rise in most European countries [1,2,3,4,5]. The raven can be a synanthropic species that can even nest in cities [6,7]. Furthermore, the raven is a food opportunist that can eat plants, prey on animals, and feed on garbage dumps. Numerous authors point to the crucial role of carrion in the species’ diet [8,9,10,11,12,13]. In Europe, ravens feed mainly on the carrion of animals killed by vehicle collisions, the game remains left by hunters, and the prey of big predators. In the vicinity of water bodies, ravens feed on dead fish, and in farmland they feed on the carrion of livestock [14,15,16,17,18,19]. 

In Central Poland, the carrion of farm animals was thought to be the main food source for ravens. For decades, numerous pig farms disposed of dead animals and animal waste illegally. This changed radically in February 2014, after the first case of African swine fever (ASF) in Poland. The first cases of the virus were reported in domestic pigs (*Sus domestica*) and wild boar (*Sus scrofa*) in Northeast Poland, and then the disease spread to the South and to the West. The closest outbreak was recorded approximately 50 km from the study area [20]. The virus is lethal to wild boar and domestic pigs, and its occurrence is a serious economic problem. Therefore, Polish veterinary services introduced actions and procedures to stop the virus from spreading. These measures included intensive controls in farms and educational activities. As a result, the illegal disposal of domestic pig remains became more difficult and carried the threat of severe fines. Moreover, the strict procedures involving the utilization of wild boar killed on the roads and the remains of hunted game were implemented [21,22,23,24]. At the same time, actions to reduce wild boar abundance were undertaken through culling and trapping in the whole of Poland. The target density of the species is from 0.1 to 0.5 individuals/km^2^ (depending on the region) [25]. Furthermore, as a part of preventive actions in areas that are under the threat of virus spread, services such as police, soldiers, and others search fields and forests for the carcasses of wild boars, which, when found, are utilized. All this has led to a severe drop in an availability of the carrion of domestic pig and wild boar carrion for ravens.

In light of these changes, the aim of this study was to assess the influence of assumed restricted access to carrion on the raven population. Therefore, we compared certain ecological aspects (i.e., population abundance, breeding parameters, and diet) before and after the implementation of restrictive procedures that are aimed at stopping the spread of ASF to Western Europe.

## 2. Materials and Methods

### 2.1. Study Area

Research was carried out in Central Poland in the area of the Experimental Forest Station of Warsaw University of Life Sciences, in the vicinity of Rogów village (51°49′6.56″ N, 19°53′4.36″ E). The study area comprised approximately 105 km^2^ of field and forest mosaic. Forests accounted for 25% of the area (approximately 2400 ha) and formed seven complexes (70–1000 ha). The main forest-forming species was Scots pine (*Pinus sylvestris*), which was dominant in 50% of the area, which was shared with oaks (*Quercus* spp.), which accounted for almost 20%, and beech (*Fagus sylvatica*) at close to 10%. The main forest habitat types were fresh mixed forest and fresh broadleaved forest (together 83%). Stands older than 60 years accounted for approximately 50% of the forest area. Stands older than 120 years formed around 2% of the forest area and were mostly protected as nature reserves. The remaining part of the area was arable land (59%), orchards (5%), grasslands (5%), and scattered buildings [26].

A few pig and poultry farms, as well as a big pond farm producing carp (*Cyprinus carpio*), were located in the area. The industry was represented by a meat processing plant and a factory of domestic animal food. The source of carrion was also from ungulates—roe deer (*Capreolus capreouls*), wild boar, red deer (*Cervus elaphus*), and fallow deer (*Dama dama*)—which were killed in this area by numerous stray dogs (*Canis familiaris*) [27]. The study area was crossed by a busy national road (not fenced) and a railway, so carrion could also be found near these infrastructures [28,29].

### 2.2. Raven Population in the Area

Data on ravens in the area come from as early as the middle of the 20th century. At that time (1945–1950), no nesting of ravens was recorded in the area [30]. However, afterwards, the population of the species started to rise. An inventory carried out during the years 1978–1992 showed five to six pairs of the species [31,32]. In the years 2001–2003, eight breeding pairs were recorded [31]. 

### 2.3. Methods

The present study was undertaken in the years 2011–2018, and this time span was divided into ‘before ASF’ (2011–2014) and ‘ASF outbreak’ (2015–2018). In each breeding season (February–May), nests of ravens were searched in the whole study area. An inventory of nests in the forest was made by two people who were equipped with binoculars and handheld transceivers, walking 150–200 m apart. Outside the forests, nests were searched from a car to check on pre-selected places, i.e., groups of high trees, high voltage poles, and other potential nesting places. All observations of the ravens were recorded on the map, and the locations of their nests were recorded with a GPS receiver (Garmin 62sc, Garmin International, Inc., Olathe, KS, USA). All located nests were checked systematically in the subsequent months. With the aid of binoculars, a number of fledglings was assessed, including the time when they left the nest. Accounting for method imperfection, nest leaving was assigned to pentads, and the onset of nest leaving was assumed to be its third day. The breeding success (percentage of pairs that successfully produced at least one fledgling) and number of fledglings were calculated, both for successful pairs and for all other known breeding pairs. 

Diet composition was assessed on the basis of an analysis of pellets (n = 958) that were collected every two weeks in the vicinity of nests (within up to several meters from the nest) from March to May. All organic and inorganic elements were separated in a laboratory; next, they were identified with the aid of a stereo microscope. Prey remains were systematically assigned with the aid of keys [33,34,35,36,37,38,39]. Furthermore, the collection of feathers, skulls, and seeds were used for comparisons. In some cases, histological analysis of hair was performed [40,41,42,43]. Finally, a proportion of pellets in which a given food category was recorded was calculated. As a complementary method, direct observations of feeding and preying ravens were conducted from March to May. Each bird that was observed to eat a certain food category was treated as a single case (overall, 531 observations). These data were collected during other field activities (also concerning other raptor studies), and no purposeful observations in selected places (e.g., garbage dumps) were made. All cases of feeding ravens were taken as 100% (each observed bird was a single case) to give the proportion of birds feeding on a certain food category. 

### 2.4. Statistical Analysis

To compare data in the two periods (before and after ASF), a Student’s *t*-test (for data with normal distribution—the number of breeding pairs) and Mann–Whitney tests (for non-normal distribution—the number of juveniles produced, the time when they left their nests) was performed. The normality of distribution was checked with the Shapiro–Wilk test. Additionally, to compare the proportion of selected food categories during the two periods, a chi-squared test was used, with Bonferroni correction for multiple comparisons applied (*p* < 0.01). A statistical analysis was performed using the Past3 software [44].

## 3. Results

In the first ‘before ASF’ period (2011–2014), on average, 12.3 (SD = 1.0) breeding pairs of ravens were recorded in the study area per year. In the second ‘ASF outbreak’ period (2015–2018), an average of 7.5 pairs (SD = 1.3) were recorded (i.e., the number of pairs dropped by 42%) (Student’s *t* test, t = 5.9, *p* < 0.005) (Table 1). The recorded densities were 11.7 pairs/100 km^2^ for the total area during the first period and 7.1 pairs/100 km^2^ for the total area during the second period. Almost all of the nests were located in forests, and nests in groups of trees or buffer strips in open landscape were recorded only three times. 

**Figure 1:**
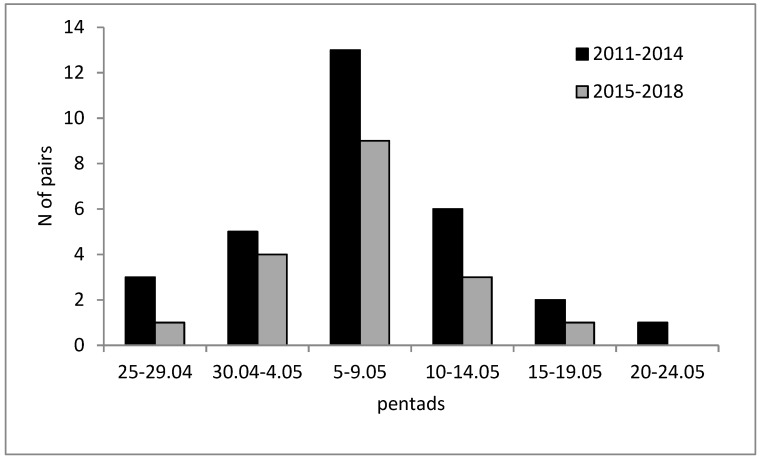
Time when juvenile ravens left their nests in the two (‘pre ASF’ and ‘ASF outbreak’) periods in Central Poland.

The breeding successes in both periods were similar (65% and 61% for the first and second periods, respectively) and the number of juveniles per breeding pair did not differ (Mann–Whitney test, Z = 1.60, *p* > 0.05). However, birds in the second period (2015–2018) had fewer fledglings per successful pair (Z = 2.89, *p* < 0.05) (Table 1). The time when juveniles left their nests (7 May, on average) did not differ between the two periods (Z = 0.35, *p* > 0.05) (Figure 1). In the first period, reasons for breeding failure were identified in 10 cases: In three cases, a tree with eggs or juveniles was cut down; in four cases, interactions between neighboring pairs of ravens were the cause of the failure; nests fell down twice; and one brood was killed by a marten (*Martes* sp.). In the second period (2015–2018), only one tree was cut down; one juvenile was found dead under the tree; two nests fell down during a storm; and one brood was killed by a white-tailed eagle (*Haliaeetus albicilla*); in the remaining cases, the reason for failure was not determined. 

A comparison of the ravens’ diet during the two periods on the basis of two methods gave similar results (Table 2 and Table 3). In both cases, a significant decrease in the share of carrion of domestic pigs and wild boar was recorded. In the years 2011–2014, domestic pigs and wild boar remains were recorded in 36.9% of the raven pellets, and in the second period they were found in 13.7% of the pellets (chi-squared test, χ^2^ = 81.59, df = 1, *p* < 0.0001) (Table 2). In the case of direct observations of birds, feeding on pig and wild boar carrion was recorded 76 times (31.3% of observations) in the first study period, but only 16 times (5.6% observations) in the second period (χ^2^ = 60.87, df = 1, *p* < 0.0001) (Table 3). At the same time, an increase of other food categories was recorded, i.e., carrion of domestic dogs and cats (*Felis catus*) was recorded in 11.2, as opposed to 3.8% of pellets (χ^2^ = 11.62, df = 1, *p* < 0.001), while remnants of small vertebrates (shrews, rodents, small birds, reptiles, and amphibians) were recorded in 75.4 as opposed to 54.8% of pellets (χ^2^ = 18.17, df = 1, *p* < 0.0001) (Table 2). In the two periods, ravens fed on roe deer and poultry carrion, fed from rubbish dumps, and made use of plant matter (Table 2 and Table 3). 

## 4. Discussion

In the ‘before ASF’ period (2011–2014) during fieldwork (both concerning this study and other done in the same area), we recorded numerous cases of illegal disposal of domestic pig carrion. The most extreme case was a dump of about 40–60 carcasses in sand pits. In other cases, between one to several carcasses were seen in one place. On the contrary, during the years 2015–2018, such cases were rare, however animal waste might have been covered with soil or garbage, and thus was more difficult to find. Additionally, due to a reduction in the wild boar population and the restrictive utilization of game remains, the availability of wild boar carrion decreased [45]. We may assume that this high rise in the raven abundance in the study area over the years 1978–2014 was possibly due to a high level of anthropogenic food availability (including carrion of farm animals). Population densities for the ‘before ASF’ period (2011–2014) were among the highest ever recorded, both in Poland and in Europe review in Reference [4]. Higher densities were shown, mostly in less extensive areas with very abundant food sources (e.g., rubbish dumps), where ravens nested in semi-colonies [3]. In Great Britain, the highest densities (up to 21 pairs/100 km^2^) were recorded in areas of intensive sheep (*Ovis aries*) production, where birds had access to the carrion of those animals [46]. Similarly, as in our case, limited access to carrion resulted in a decrease in raven abundance in those areas [46,47]. Population decreases were also recorded in Israel and in the Canary Islands, mainly due to farming transformation and pesticide usage [1]. Research carried out in a primeval life environment for the raven, Białowieża National Park, showed similar densities to those recorded in our case for the period 2011–2014. This high density was attributed to high forest cover and high carrion availability, i.e., the remains of prey killed by wolves (*Canis lupus*) and lynxes (*Lynx lynx*) [18,48]. 

We need to keep in mind that numerous possible factors (other than a decrease in carrion availability) might have been responsible for the population decrease observed in our study. However, landscape characteristics (i.e., forest cover, the share of built-up areas) did not change in the two study periods. No apparent change in waste disposal took place in the two periods. In the study area, there are numerous illegal small rubbish dumps, however they are not very large or permanent. While, in the long term, winters became milder in Central Poland [49], this change was not so rapid as to have an important influence on the population abundance over the eight-year period. Ravens have not been persecuted in the study area, as this is a hunting ground of the Experimental Forest Station of Warsaw University of Life Sciences. Moreover, despite heavy penetration of forests, in the course of numerous field studies carried out in the area, no cases of dead ravens killed by disease or poisoning were reported. Finally, the population of predators (in this case stone marten (*Martes foina*) and pine marten (*M. martes*)) remained stable [50].

The situation observed in our study area is relatively new, so very few data is available from other areas in Poland for comparisons. In general, the raven population trend is stable in Poland (nonetheless, regional variation is found) [5]. The only new data come from Sobibór Forest (25,695 ha) in Eastern Poland. The raven population was estimated to be closer to that observed in the ‘before ASF’ period in our study (9.3–12.7 pairs/100 km^2^ in 2016–2017), but was assumed to be stable in the last 30 years [51]. While that study did not focus on raven diet, we may expect that birds inhabiting big, continuous forest complex did not rely on anthropogenic food.

Limited access to the carrion of domestic pig and wild boar in our study did not influence the breeding success of the raven population, which was around 60% in the two periods. In other European studies, reproductive success varied from 42% in the Shetland Islands [15] to over 90% in Western Poland [52]. The latter case involved populations nesting on high-voltage poles in an open landscape. The juvenile production per breeding pair did not differ between the studied periods, however was slightly lower per successful pair in the second period. Nevertheless, those parameters did not differ much from the results of other European populations [18,46,53]. The highest productivities per breeding pair and per successful pair, 3.3 and 4.1 juveniles, respectively, were recorded in Denmark review in Reference [4]. Limited access to carrion in our study did not influence the time when juveniles left their nests, and in both periods birds started to lay eggs at the turn of February and March. Nevertheless, many authors point to the relationship between food availability and the onset of egg-laying in birds [54,55,56]. 

A similar case of changes in carrion availability and its influence on scavenger population was reported in Western Europe, where abundant carcasses of domestic animals permitted the existence and growth of huge vulture populations. However, from 2000 onwards, European Union (EU) sanitary legislation due to the appearance of bovine spongiform encephalopathy progressively limited the abandonment of dead animals in the field, resulting in a sudden reduction of food availability. This influenced the diet of some vulture species (i.e., griffon vulture (*Gyps fulvus*)), which consumed significant amounts of wild rabbits (*Oryctolagus cuniculus*) and garbage as a result [57]. Moreover, a decrease in breeding success, an increase in mortality in young age classes, an increase in the number of cases of vultures attacking and killing cattle, and a halt in population growth, were observed in vulture population [58]. The case of the Pyrenean bearded vulture (*Gypaetus barbatus*) population, showed a delay in laying dates and a regressive trend in clutch size, breeding success, and survival, following this policy change [59].

The relationship between humans and scavengers can be mutualistic. For example, in Socotra (Yemen) human activities enable the maintenance of the densest population of Egyptian vulture (*Neophron percnopterus*), whereas vultures provide a key regulating service by disposing of up to 22.4% of the organic waste produced annually [60]. Therefore, the decline of scavengers has a range of socioeconomic, as well as cultural and biodiversity, impacts [61]. One of the most striking of these is the human health impacts of the vulture decline [61]. In India, the accumulation of livestock carcasses (following a drop in vulture population) led to apparent increases in feral dog and rat (*Rattus* sp.) populations [61,62]. This also poses a serious threat to human and domestic animal health, as uneaten carcasses become infested with potentially pathogenic bacteria, e.g., anthrax, while increasing populations of dogs and rats serve as reservoirs and vectors of diseases such as canine distemper virus, canine parvovirus, *Leptospira* spp. bacteria, and rabies [61,62,63].

Our results confirm that the raven is a food opportunist and that carrion plays an important role in its diet. Nevertheless, it needs to be considered that the real share of carrion in the species’ diet could have been even higher than was confirmed by pellet analysis. Probably, in most cases, eaten meat was totally digested, and no remains, such as feathers or fur, were found in pellets. This especially applies to chicken remains that come from poultry processing plants (it needs to be pointed out that the strict procedures applied to pigs do not apply to poultry carrion, so that this was available in the two periods). It is different in the case of prey killed by ravens, e.g., small vertebrates or insects, where we may expect to find some remains in a pellet from each eaten individual. The same applies to fruit, e.g., cherries or plums. In our case, the carrion of domestic pigs was found to be the most important food item, and when its supply was limited, ravens needed to modify their feeding strategies, i.e., they fed on the carrion of domestic dogs and cats and preyed on small vertebrates more often. Free-roaming farm dogs and cats are very numerous in the study area [27,64], and they can get killed on the road or on the railway or their carcasses can be disposed of when they die. It should also be stressed that, in our case, ravens fed on carrion during the breeding season, which points to its crucial role in the population condition. In other areas, a high share of carrion in the raven diet was recorded only in autumn and winter [65,66,67].

## 5. Conclusions

In our study the carrion of farm animals (disposed of illegally) was the main food source for ravens in the past. Its high availability allowed local population to reach one of the highest densities ever recorded. With the introduction of strict procedures for the utilization of pig and wild boar carcasses to stop the spread of the ASF virus, thus drop in the carrion availability, ravens changed their diet, i.e., they fed on the carrion of dogs and cats or preyed on small vertebrates more often. As a result raven population abundance decreased rapidly. Overall, our study points to a crucial role of the availability of the carrion of big farm animals (i.e., domestic pig) in maintaining the high density of breeding raven populations in a human-transformed habitat such as field and forest mosaic.

## Figures and Tables

**Table 1:** Breeding parameters of the raven population in Central Poland in the ‘pre ASF’ (2011–2014) and ‘ASF outbreak’ (2015–2018) periods.

Year/Period	N of Breeding Pairs	Breeding Success (%)	N juv. per	
			Breeding Pair	Successful Pair
2011	13	61	1.5	2.5
2012	13	61	1.5	2.5
2013	11	63	1.6	2.6
2014	12	75	1.7	1.7
$\bar{x}$ (SD)	12.3 (1.0)	65.0	1.6 (1.3)	2.3 (0.7)
2015	8	87	1.8	2.0
2016	6	33	0.8	2.5
2017	7	71	1.1	1.6
2018	9	55	0.9	1.6
$\bar{x}$ (SD)	7.5 (1.3)	61.5	1.2 (1.7)	1.9 (0.7)

**Table 2:** Dietary composition of ravens in Central Poland in the two (‘pre ASF’ and ‘ASF outbreak’) periods on the basis of pellet analysis.

Food Category	2011–2014		2015–2018	
	Pellets		Pellets	
	N	%	N	%
Domestic pig	156	32.9	56	11.6
Wild boar	19	4.0	10	2.1
Domestic cow	5	1.1		0.0
Domestic dog	14	3.0	39	8.1
Domestic cat	4	0.8	15	3.1
Domestic rabbit	2	0.4		0.0
Moose	3	0.6		0.0
Roe deer	27	5.7	49	10.1
Racoon dog	3	0.6		0.0
Red fox	1	0.2	4	0.8
*Mustela* sp.	1	0.2		0.0
Brown hare	1	0.2		0.0
Soricomorphs	58	12.2	90	18.6
Small rodent	78	16.5	140	28.9
Chicken	95	20.0	116	24.0
Domestic pigeon	2	0.4	5	1.0
Small bird	43	9.1	82	16.9
Eggs	34	7.2	62	12.8
Fish	54	11.4	63	13.0
Amphibians	15	3.2	29	6.0
Reptiles	18	3.8	24	5.0
Invertebrates	258	54.4	329	68.0
Grains	268	56.5	300	62.0
Fruits	44	9.3	58	12.0
Unrecognized plants	135	28.5	169	34.9
Inorganic elements	139	29.3	183	37.8
Total N of pellets	474		484	

**Table 3:** Dietary composition of ravens in Central Poland in the two (‘pre ASF’ and ‘ASF outbreak’) periods on the basis of direct observations of feeding birds.

Food Category	2011–2014		2015–2018	
	Observations		Observations	
	N	%	N	%
Domestic pig	66	27.2	14	4.9
Wild boar	10	4.1	2	0.7
Domestic dog	2	0.8	6	2.1
Domestic cat	2	0.8	3	1.0
Roe deer	9	3.7	21	7.3
Small rodent	4	1.6	9	3.1
Mole	2	0.8	6	2.1
Chicken	54	22.2	85	29.5
Small birds	1	0.4	2	0.7
Eggs	2	0.8	1	0.3
Fish	3	1.2	6	2.1
Insects	6	2.5	10	3.5
Earthworm	4	1.6	8	2.8
Plant food	41	16.9	60	20.8
Garbage	20	8.2	34	11.8
Industrial waste	12	4.9	15	5.2
Excrements	5	2.1	6	2.1
Total N of observations	243	100	288	100
